# An Unusual Cause of Chronic Back Pain: Solid Pseudopapillary Neoplasm of the Pancreas

**DOI:** 10.7759/cureus.111280

**Published:** 2026-06-22

**Authors:** Mutaz Kalas, Shivangini Duggal, M Ammar Kalas, Sherif E Elhanafi

**Affiliations:** 1 Internal Medicine, Texas Tech University Health Sciences Center, El Paso, USA; 2 Gastroenterology and Hepatology, The University of Texas at Tyler, Tyler, USA

**Keywords:** atypical back pain, pancreatic neoplasm, radicular low back pain, solid pseudo-papillary tumor of the pancreas, spn

## Abstract

Solid pseudopapillary neoplasms (SPNs) are rare, low-grade malignant tumors of the pancreas comprising 1%-2% of pancreatic neoplasms. Predominantly affecting young women, these tumors are typically slow-growing and often discovered incidentally during imaging for non-specific symptoms. This case describes a young female patient who presented with acute-onset back pain without prior significant medical history or trauma. Initial evaluation suggested a musculoskeletal etiology; however, persistent symptoms prompted further imaging, which revealed a pancreatic mass. Subsequent diagnostic workup, including cross-sectional imaging and histopathological analysis, confirmed the diagnosis of an SPN of the pancreas. The patient underwent surgical resection with a favorable postoperative recovery. This case highlights the atypical presentation of SPN as isolated back pain and underscores the importance of maintaining a broad differential diagnosis when evaluating persistent, unexplained pain in young patients.

## Introduction

Solid pseudopapillary neoplasms (SPNs) are rare, low-grade malignant tumors of the pancreas that predominantly affect young women in their second or third decade of life. They account for 1%-2% of all exocrine pancreatic neoplasms in adults, and approximately 5% of all cystic pancreatic neoplasms [1]. They are often discovered incidentally on imaging or present with non-specific symptoms, such as abdominal pain, nausea, or back discomfort [2].

SPNs of the pancreas are often first detected using ultrasonography, where they appear as well-defined hypoechoic cystic masses with limited internal vascularity [3]. Contrast-enhanced ultrasonography can highlight an enhanced capsule and a non-enhanced central area, though its diagnostic specificity is limited [4]. Computed tomography (CT), particularly multi-detector CT (MDCT), is the most widely used and sensitive imaging modality for evaluating SPNs, offering a detailed assessment of lesion morphology and resectability. Typical CT features include a well-circumscribed solid-cystic mass, peripheral and fibrous enhancement, and signs of hemorrhage or calcification [5]. Magnetic resonance imaging (MRI) provides superior soft tissue contrast and reveals classic features such as T2 hyperintensity and heterogeneous enhancement on contrast-enhanced T1-weighted sequences [6]. Endoscopic ultrasound (EUS) allows for high-resolution imaging and tissue acquisition. While EUS-guided fine needle aspiration (EUS-FNA) has good sensitivity and specificity (>80%) [7], EUS-guided fine needle biopsy (EUS-FNB) offers superior diagnostic yield due to larger tissue acquisition, enabling immunohistochemical profiling. Focal uptake of fluorodeoxyglucose (FDG) is commonly observed in SPNs, indicating increased glucose metabolism. In cases where FDG uptake is absent, newer tracers such as fibroblast activation protein inhibitor (FAPI) may help identify tumors due to their affinity for stromal fibroblast activity [8]. Overall, EUS-based biopsy remains the most accurate modality for preoperative diagnosis [9].

Histopathologic evaluation often reveals pseudopapillary architecture, cytoplasmic vacuoles, and eosinophilic hyaline globules [10]. Immunohistochemistry staining is typically positive for vimentin, synaptophysin, CD10, progesterone receptor, and β-catenin [11]. Surgical resection is the mainstay of treatment and offers a favorable prognosis, with a five-year survival rate of approximately 97%. Although they have a low potential for malignancy, the metastatic rate is approximately 10%-15% [12]. This case highlights the importance of considering SPNs in the differential diagnosis of pancreatic masses, emphasizing the critical role of early detection and prompt surgical intervention in achieving favorable clinical outcomes.

## Case presentation

A 23-year-old girl with no past medical history presented with a two-day duration of severe acute sharp lower back pain radiating to bilateral flanks, followed by three episodes of non-bilious emesis. The patient was hemodynamically stable, with unremarkable laboratory work, including complete blood count, metabolic panel, and pancreatic lipase levels. She denied any history of trauma or recent falls. Physical examination was also unremarkable, with no abdominal or back tenderness noted. A previous CT abdomen performed seven years ago, obtained during evaluation for similar symptoms, including a one-day history of progressive sharp back pain, identified a 15 mm well-defined hypodense lesion in the pancreatic tail. At that time, further characterization with abdominal MRI was recommended, and the differential diagnosis included pancreatic adenocarcinoma, pancreatic neuroendocrine tumor, SPN, and pancreatic blastoma. However, the patient was subsequently lost to follow-up, and the additional imaging was not performed. Given persistent symptoms, a non-contrast CT of the abdomen (Figure 1) was performed, which demonstrated a mass in the tail of the pancreas measuring 5.7 x 4.7 x 4.2 cm.

**Figure 1:**
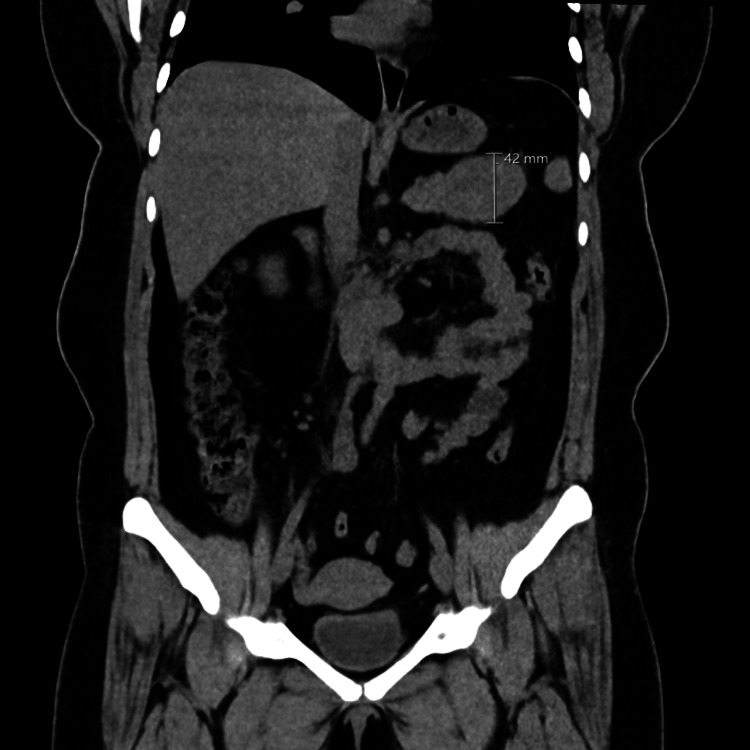
Computed tomography (CT) abdomen Mass in the tail of the pancreas measuring 5.7 x 4.7 x 4.2 cm.

The patient subsequently underwent abdominal MRI and EUS. MRI abdomen (Figure 2) revealed a 4.7 x 5.1 cm solid mass within the tail of the pancreas, without evidence of lymphadenopathy, hepatic lesions, or ductal dilation.

**Figure 2:**
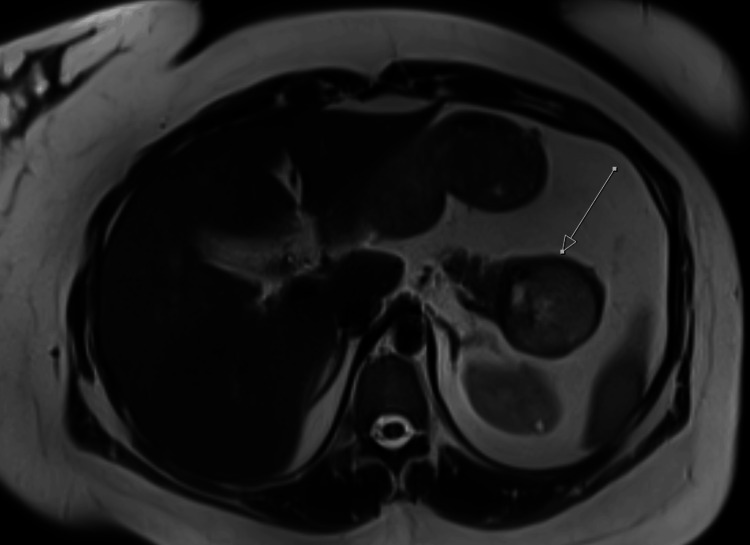
Magnetic resonance imaging (MRI) abdomen 4.7 x 5.1 cm solid mass within the tail of the pancreas, without evidence of lymphadenopathy, hepatic lesions, or ductal dilation.

Upper EUS (Figure 3) demonstrated a hypoechoic and heterogeneous mass measuring 50 x 47 mm in the maximal cross-sectional diameter with no other ultrasonographic abnormalities.

**Figure 3:**
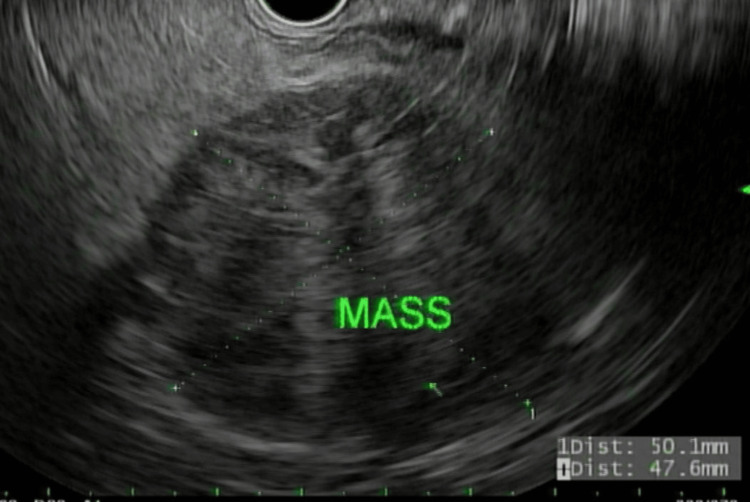
Endoscopic ultrasound (EUS) Hypoechoic and heterogeneous mass measuring 5.0 x 4.7 cm.

An FNB was performed using a 22-gauge biopsy needle using a trans-gastric approach. Histopathologic analysis demonstrated focal synaptophysin expression (Figure 4), nuclear beta-catenin expression (Figure 5), CD10 expression, and loss of E-cadherin (Figure 6), findings consistent with a diagnosis of SPN.

**Figure 4:**
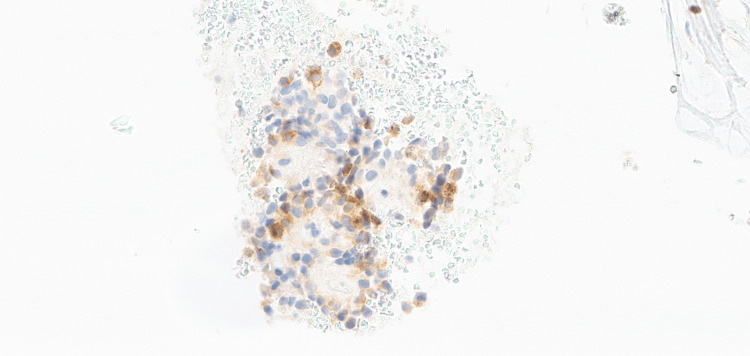
Synaptophysin immunohistochemical stain Tumor cells showing focal synaptophysin expression, original magnification x400.

**Figure 5:**
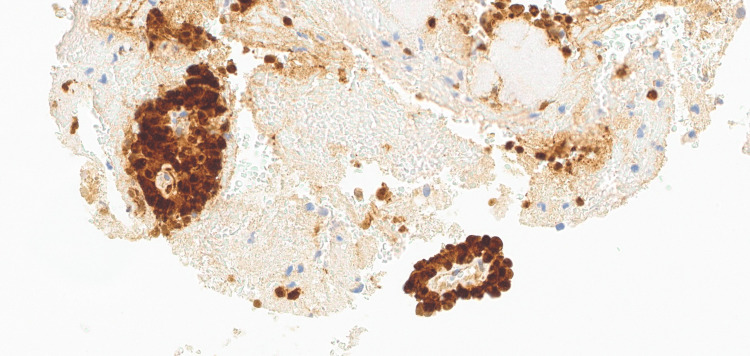
Beta-catenin immunohistochemical stain Tumor cells displaying strong nuclear expression for beta-catenin, original magnification x400.

**Figure 6:**
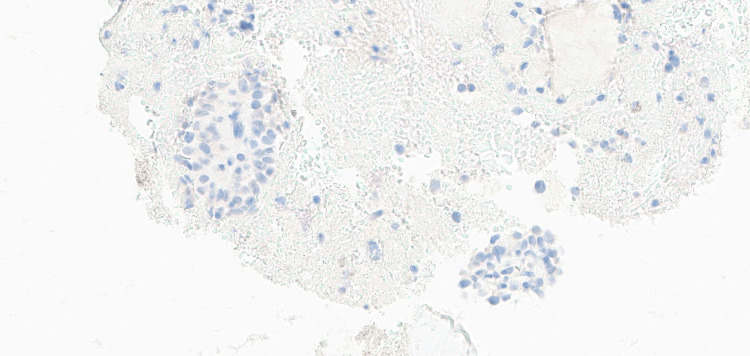
E-cadherin immunohistochemical stain Tumor cells displaying loss of E-cadherin, original magnification x400.

Biopsy specimens confirmed the presence of SPN without evidence of metastatic disease. Following these findings, the patient elected to proceed with surgical resection. A robotic distal pancreatectomy, splenectomy, and partial omentectomy were performed, and the patient was safely discharged following an uneventful postoperative course. Notably, the patient’s back pain significantly improved and subsequently subsided after resection, supporting a likely association between the presenting symptom and the pancreatic lesion. The surgical pathology report confirmed an SPN measuring 5 cm confined to the pancreas; 17 lymph nodes were negative for malignancy, and the omentum and spleen were unremarkable. The patient was discharged with outpatient follow-up in heme-oncology, gastroenterology, and surgery.

## Discussion

This case highlights a rare presentation of SPN manifesting as acute back pain, with an indolent clinical course spanning six years. Due to advances in diagnostic techniques and increased awareness, the incidence of SPNs has increased. Approximately 80%-90% of cases affect women, most often in their second or third decade of life. However, they can be seen in the pediatric and older population. In adults, they are most often located in the body or tail, whereas in pediatric patients, they are most often found in the head of the pancreas [11]. Usually, these tumors are found incidentally on imaging done for non-specific symptoms such as abdominal pain. The most reported symptom is abdominal discomfort at 36.6%, followed by nausea, vomiting, and back pain [13]. Our patient presented with the chief complaint of acute lower back pain, a rare presentation. Similarly, our patient's mass was incidentally found in 2018 and located in the tail of the pancreas. Fortunately, it remained relatively stable for seven years, with no signs of local invasion until her resection in 2025, demonstrating the slow growth of these tumors. At the time of admission, laboratories and tumor markers such as CEA and CA-19-9 are generally unremarkable. A CT thorax was negative for metastasis. The patient underwent EUS with FNA and was discharged with no complications. She was referred to surgical oncology for resection and seen in outpatient gastroenterology for follow-up, and she reported no new symptoms. SPNs have a slight potential for malignant transformation of around 15%, making surgical resection the mainstay of treatment [11]. Close outpatient follow-up after resection is essential in monitoring for recurrence. Currently, there is no literature to support the use of systemic anti-cancer therapies such as chemotherapy and radiation.

An SPN diagnosis relies on a combination of imaging, followed by EUS or endoscopic retrograde cholangio-pancreatography (ERCP), as well as histopathological and immunohistochemical evaluation. FNB and FNA are often done to guide the surgical approach. At diagnosis, the average size of SPNs varies; the reported mean diameters range from 4.4 to 9.03 cm [14]. Our patient’s mass fell into the average range, and the MRI abdomen was notable for a 4.7 x 5.1 cm mass in the pancreatic tail. There was no associated adenopathy, ductal dilation, or hepatic lesions. On CT, SPNs appear as significant, well-circumscribed, heterogeneous masses and often lack vascularity. To provide better visualization, an MRI can highlight the cystic portions and hemorrhage often found within the tumor. However, our patient's MRI reported a solid mass without cystic portions. Despite these tumors being relatively large, it is rare for vascular occlusion or encasement to occur. A handful of demographic and imaging features can highly suggest SPN; however, no statistically significant features can predict clinically aggressive behavior [15].

The surgical approach differs based on the location of the tumor. Postoperative complications are common, occurring in up to 30% of patients. The most frequent being a pancreatic fistula and surgical site infection [16]. For a tumor located on the tail of the pancreas, a laparoscopic distal pancreatectomy is preferred because it does not need manual anastomosis or reconstruction of the digestive tract. Preserving the spleen in distal pancreatectomy is up to the surgeon's discretion based on the patient's histopathological features and surgical anatomy. Moreover, laparoscopic resection is the gold standard and is associated with lower morbidity and mortality than open surgery. Overall prognosis is favorable, and the five-year survival rate is about 97% [11]. Malignant transformation is observed in 10%-15% of cases, with metastasis most frequently found in the liver and peritoneum. Our patient underwent robotic distal pancreatectomy, partial omentectomy, and splenectomy, with vaccine prophylaxis given. The surgical pathology report was remarkable for a 5 cm solid-pseudopapillary neoplasm with no evidence of lymph-vascular or perineural invasion. In conclusion, SPNs are rare, low-grade pancreatic tumors that primarily affect young women and, with surgical resection, have a favorable prognosis.

## Conclusions

This case underscores the importance of maintaining SPN within the differential diagnosis of pancreatic masses, particularly in young patients presenting with atypical or non-specific symptoms. It further highlights the rare presentation of SPN as isolated back pain and the potential for a prolonged indolent clinical course that may delay definitive diagnosis. Together, these features emphasize the need for sustained clinical vigilance, timely cross-sectional imaging, and prompt surgical management to optimize outcomes in this uncommon pancreatic neoplasm.
